# Skin decontamination in critically ill patients. Comparison of two daily bathing methods: traditional bath versus 2% chlorexidine gluconate (CHG) cloths

**DOI:** 10.1186/2047-2994-4-S1-P239

**Published:** 2015-06-16

**Authors:** G Tura, N Marcatelli, P Leardini

**Affiliations:** 1 Rischio Infettivo, Rimini, Italy; 2 Intensive care unit, Ausl Romagna AOO Rimini, Rimini, Italy

## Introduction

Healthcare Associated Infections (HAI) in Intensive Care Units (ICU) settings are still a challenge; skin decontamination is a recommended intervention to interrupt pathogen transmission through “source control”^1^. Various studies^1,2,3,4^ investigated the effectiveness of 2 % CHG cloths compared with the use of traditional bathing (soap and water) or pre - impregnated cloths with soap solution (no antiseptic) for the reduction of MDROs acquisitions and blood-stream infections in Intensive Care Unit settings.

## Methods

The system used for daily patient hygiene at the General Intensive Care Unit (ICU) of the Rimini hospital until April 2013 consisted of a “traditional” method (water and soap). Since May 2013, 2 % CHG cloths (Sage Products LLC) were introduced for the daily bathing of colonized/infected patients (targeted decolonization)^3^. Afterwards, since October 2013, a new procedure was introduced to extend daily bathing with 2 % CHG cloths to all patients in the ICU (universal decolonization)^3^. Patients between periods had similar characteristics.

## Results

Results compare two periods in which data were collected and analyzed: Period A - November 2012 / April 2013 - 6 months of daily hygienewith soap and water - Pre - intervention.

Period B - October 2013 / July 2014 - 10 months of daily hygiene with 2 % CHG cloths (universal decolonization) Intervention. Number of patients Colonized/Infected: decreased by 51.21% (p - value = 0.00141). Positive blood cultures decreased by 68.32 % (p - value = 0.01699) Return on investment(ROI): estimated decrease of 50% of the total costs, intervention period vs. pre-intervention period.

## Conclusion

Daily bathing with 2% CHG cloths significantly reduces the number of patients colonized/infected and MDRO acquisitions. The results and the estimated ROI obtained at the General Intensive Care Unit(ICU) of the Rimini hospital, confirm the opportunity of implementing a universal decolonization protocol in ICU settings.

## Disclosure of interest

None declared.

